# High-Intensity Interval Training Improves Functional Capacity and Peripheral Skeletal Muscle Energy Metabolism in Infarcted Rats

**DOI:** 10.3390/antiox15101352

**Published:** 2026-10-19

**Authors:** Thierres H. D. Pontes, Gabriela Brandao, Eder A. Rodrigues, Ricardo C. Damatto, Lidiane M. Souza, Mariana J. Gomes, Felipe C. Damatto, Luana U. Pagan, Élida P. B. Ojopi, Anna C. C. Santos, Ana P. S. Marreiros, Gilson M. Murata, Leonardo A. M. Zornoff, Katashi Okoshi, Marina P. Okoshi

**Affiliations:** 1 Department of Internal Medicine, Botucatu Medical School, Sao Paulo State University—UNESP, Botucatu 18618-687, Brazil; thierres.pontes@unesp.br (T.H.D.P.); gabriela.brandao@unesp.br (G.B.); eder.rodrigues@unesp.br (E.A.R.); ricardo.damatto@unesp.br (R.C.D.); lidiane.souza@unesp.br (L.M.S.); felipe.damatto@unesp.br (F.C.D.); luana.pagan@unesp.br (L.U.P.); benquique.ojopi@unesp.br (É.P.B.O.); anna.consorti@unesp.br (A.C.C.S.); ana.marreiros@unesp.br (A.P.S.M.); leonardo.zornoff@unesp.br (L.A.M.Z.); katashi.okoshi@unesp.br (K.O.); 2 Department of Kinesiology and Sport Management, Texas A&M University, College Station, TX 77845, USA; m.janinigomes@tamu.edu; 3 Laboratory of Medical Investigation (LIM-29), Clinic Medical Department, University of Sao Paulo Medical School, Sao Paulo 05360-160, Brazil; gilson.murata@usp.br

**Keywords:** exercise, cardiac remodeling, myocardial infarction, rat, soleus muscle, enzyme activity

## Abstract

Introduction: High-intensity interval training (HIIT) has been recommended in post-myocardial infarction (MI) cardiac rehabilitation; however, its effects on MI-induced cardiac remodeling and skeletal muscle remain unclear. This study evaluated the effects of HIIT on functional capacity, cardiac remodeling, and the soleus skeletal muscle in rats after long-term MI. Methods: Three months after MI induction, Wistar rats were assigned to Sham, MI, and MI subjected to HIIT (MI-HIIT) groups. HIIT was performed three times per week for three months on a treadmill. Cardiac function and functional capacity were assessed before and after training. Oxidative stress markers, energy metabolism enzyme activities, and trophism were analyzed in the soleus. The statistical analysis included ANOVA and Bonferroni or Kruskal–Wallis and Dunn tests. Results: HIIT improved functional capacity compared to the Sham and MI groups. The infarcted groups had left ventricular dilation, hypertrophy, and systolic dysfunction, which were not modulated by HIIT. Soleus fiber cross-sectional areas did not differ between the groups. Catalase activity was lower in the MI and MI-HIIT groups than in the Sham group, while other oxidative stress markers were unchanged. HIIT increased citrate synthase activity compared to the Sham and MI groups, and phosphofructokinase and carnitine palmitoyltransferase-1 activities compared to the MI group. Conclusions: High-intensity interval training improves functional capacity despite unchanged cardiac remodeling in long-term infarcted rats. Enhanced physical capacity is associated with the increased activity of the enzymes involved in the energy metabolism of the soleus muscle.

## 1. Introduction

Heart failure has a significant impact on morbidity and mortality [1]; it is a progressive clinical syndrome resulting from structural and/or functional cardiac abnormalities that lead to pulmonary or systemic congestion and low cardiac output [2]. Despite major advances in cardiovascular therapies over recent decades, heart failure remains frequently caused by myocardial infarction (MI) [3,4]. After MI, molecular, cellular, and interstitial cardiac changes occur and manifest clinically as alterations in cardiac mass, geometry, and function, potentially leading to ventricular dysfunction and heart failure [5]. The process is known as cardiac remodeling.

Heart failure is associated with exercise intolerance, characterized by dyspnea, fatigue, and reduced ability to perform physical activities. In addition to cardiac dysfunction, skeletal muscle abnormalities contribute to the clinical manifestations of heart failure. Since the 1990s, abnormalities in peripheral skeletal muscles have been described during cardiac remodeling and heart failure [6,7]. Muscle alterations include structural changes, such as atrophy, fibrosis, and reduced capillary density; metabolic, enzymatic, and biochemical alterations; increased oxidative stress; mitochondrial dysfunction; and inflammation [8,9].

International guidelines recommend physical exercise as a non-pharmacological therapeutic strategy for heart failure patients in functional classes I to III of the New York Heart Association classification to improve quality of life and reduce hospitalization [10]. High-intensity interval training (HIIT) is characterized by alternating short periods of exercise performed at ≥80% of peak oxygen uptake (VO_2_peak) with periods at 40–50% of VO_2_peak [11,12,13]. Studies have shown the superiority of HIIT over continuous moderate aerobic exercise in enhancing VO_2_peak, quality of life, and functional capacity in patients with coronary artery disease [14,15,16,17,18]. We have previously observed that early HIIT improves cardiac energetic metabolism and decreases myocardial oxidative stress in post-MI rats [19]. Very few authors have evaluated the effects of HIIT on skeletal muscle from infarcted rats [20].

In this study, we tested the hypothesis that HIIT-induced increase in functional capacity is combined with improvements in peripheral skeletal muscle parameters. To achieve this, we evaluated the effects of high-intensity interval training on functional capacity, cardiac remodeling, and peripheral skeletal muscle in rats following long-term myocardial infarction. Muscle trophism, the activity of enzymes involved in energy metabolism, and oxidative stress markers were analyzed in the soleus muscle.

## 2. Material and Methods

### 2.1. Experimental Design

Male Wistar rats obtained from the Animal Facility at Sao Paulo State University (UNESP), Campus of Botucatu, were fed a commercial Purina^®^ diet and water ad libitum, and housed in a room with controlled temperature and humidity and an inverted light–dark cycle. All experimental procedures were approved by the Ethics Committee on Animal Use of Botucatu Medical School, UNESP (protocol n° 1236/2017—CEUA).

Acute MI was induced after anesthesia with ketamine hydrochloride (60 mg/kg) and xylazine (10 mg/kg) by ligation of the left anterior descending coronary artery. The control group (Sham) rats were subjected to the same surgical procedure without coronary artery ligation. Three months after surgery, all of the rats were subjected to transthoracic echocardiography, an exercise tolerance test, and subsequently allocated into three groups: Sham (n = 20); myocardial infarction (MI; n = 26); and MI submitted to high-intensity interval training (MI-HIIT; n = 24). At the end of the experimental period, the rats underwent the same evaluations. The following day, 72 h after the last training session, the animals were weighed, anesthetized with sodium pentobarbital (50 mg/kg IP), and euthanized.

Two examiners evaluated the rats for in vivo clinical evidence of heart failure—specifically tachypnea or labored breathing. Post-mortem examination involved screening for ascites, pleural and pericardial effusion, atrial thrombi, right ventricular hypertrophy (right ventricular weight [mg]/body weight [g] ratio > 0.8), and pulmonary congestion (a lung weight/body weight ratio > 2 standard deviations above the control group mean).

### 2.2. Exercise Tolerance Test

Functional capacity was analyzed by an exercise tolerance test performed before the training period, 45 days after its initiation (for adjusting exercise intensity), and at the end of the experiment. The animals were acclimated to the testing environment for one week prior to the evaluations for 5 min/day. The test consisted of treadmill running, starting at 5 m/min, with increments of 3 m/min every 3 min, until animal exhaustion. The maximum running speed was recorded, and the total distance covered was calculated [21].

### 2.3. Exercise Protocol

The exercise protocol began three months after MI induction and lasted three months, using a treadmill three times per week on alternate days. An adaptation period consisted of a gradual increase in running speed and exercise duration, as shown in Table 1. From the sixth week of training onward, the animals underwent training sessions of 3 min at 60% of maximal exercise capacity (defined as the highest speed achieved during the exercise tolerance test), followed by 4 min at 85% of maximal capacity. The sequence was repeated seven times, totaling 49 min of training per session.

### 2.4. Echocardiography

Cardiac structures and left ventricular (LV) function were evaluated before the exercise protocol to ensure homogeneity between sedentary and exercised groups, and 48 h after the last training session by transthoracic echocardiography. The rats were anesthetized with ketamine hydrochloride (50 mg/kg IP) and xylazine hydrochloride (1 mg/kg IP). Examinations were performed using a General Electric Medical Systems ultrasound (Vivid S6, Tirat Carmel, Israel) equipped with a multifrequency transducer operating at 5–11.5 MHz by the same researcher (K.O.). Mitral and aortic transvalvular flow assessments were performed using the same transducer operating at 5.0 MHz. The variables were analyzed as previously described [22].

### 2.5. Histological Analysis of the Heart

Ring-shaped LV samples were sectioned 6 mm from the apex toward the base, fixed and stained with hematoxylin and eosin. Morphometric assessment was performed using a LEICA DM LS microscope coupled to a video camera, which transmitted digital images to a computer equipped with image analysis software (Image-Pro Plus, version 7.0, Media Cybernetics, Silver Spring, MD, USA). The infarct size was calculated by dividing the sum of the epicardial and endocardial lengths of the infarcted segment by the sum of the total epicardial and endocardial circumferences; the values were expressed as a percentage of total LV area.

### 2.6. Histological Analysis of Skeletal Muscle

The soleus skeletal muscle samples were sectioned using a cryostat at −20 °C and stained with hematoxylin and eosin. Muscle fiber cross-sectional areas were evaluated using a light microscope. Between 150 and 200 muscle fibers from each rat were measured. All histological analyses were conducted using ImageJ software (version 1.51r, National Institutes of Health, Bethesda, MD, USA) by a single examiner blinded to the experimental groups.

### 2.7. Analysis of Antioxidant Enzyme Activity and Lipid Hydroperoxide Levels

Approximately 100 mg of soleus samples were homogenized in a Potter–Elvehjem homogenizer in 2 mL of 0.1 M sodium phosphate buffer (pH 7.0). The homogenates were centrifuged at 10,000 rpm for 15 min at 4 °C, and the supernatants were collected for determination of the total protein concentration, lipid hydroperoxide concentration, and the activities of the antioxidant enzymes superoxide dismutase (SOD), glutathione peroxidase (GSH-Px), and catalase [23]. Spectrophotometric measurements were performed using a Pharmacia Biotech spectrophotometer (Pharmacia Biotech, Uppsala, Sweden) with Swift II software, version 2.06 (Swift Optical Instruments, Cambridge, UK) and a microplate reader (μQuant MQX200; BioTek Instruments, Winooski, VT, USA) equipped with KCjunior software, version 1.4 (Bio-Tech Instruments, Winooski, VT, USA). All reagents were obtained from Sigma-Aldrich (St. Louis, MO, USA).

Glutathione peroxidase (GSH-Px; EC 1.11.1.9) activity was determined based on the oxidation of glutathione in the presence of hydrogen peroxide and cumene hydroperoxide. Superoxide dismutase (SOD; EC 1.15.1.1) activity was determined based on the inhibition of nitroblue tetrazolium (NBT) reduction by superoxide radicals generated from reduced nicotinamide adenine dinucleotide (NADH) and phenazine methosulfate under physiological pH conditions. Catalase (CAT; EC 1.11.1.6) activity was determined in phosphate buffer at pH 7.0, using 12.5 μL of the sample and 30% hydrogen peroxide. Spectrophotometric measurements for catalase activity were performed at 240 nm.

The lipid hydroperoxide concentration was determined based on the oxidation of Fe^2+^ supplied as ammonium ferrous sulfate. The Fe^3+^ generated during the reaction reacts with xylenol orange to form a colored complex, which was quantified spectrophotometrically at 560 nm.

### 2.8. Assessment of Energy Metabolism Enzymes

Energy metabolism was assessed by measuring the maximal activities of enzymes involved in glucose metabolism [phosphofructokinase (PFK; EC 2.7.1.11) and pyruvate kinase (PK; EC 2.7.1.40)], anaerobic metabolism [lactate dehydrogenase (LDH; EC 1.1.1.27)], the tricarboxylic acid cycle and aerobic metabolism [citrate synthase (CS; EC 4.1.3.7)], fatty acid oxidation [carnitine palmitoyltransferase 1 (CPT1; EC 2.3.1.21)], and the phosphagen energy system [creatine kinase (CK)] [24].

The soleus muscle samples (~30 mg) were homogenized in 50 mM Tris–HCl buffer containing 1 mM EDTA and protease inhibitor (Sigma, Ref. S8820-2TAB), pH 7.4, using 0.5-mm zirconium beads for 5 min at 4 °C in a Bullet Blender homogenizer. The homogenate was centrifuged at 12,000 rpm for 10 min at 4 °C, and the supernatant was collected. Enzymatic activities were determined at 25 °C, using a SpectraMax 250 microplate spectrophotometer (Molecular Devices, Sunnyvale, CA, USA).

PFK activity was determined using an assay mixture containing Tris-HCl, 2 mM MgSO_4_, 5 mM KCl, 0.2 mM NADH, 1 mM ATP, 3 mM fructose-6-phosphate (F6P), 2 mM phosphoenolpyruvate (PEP), 2 U lactate dehydrogenase (LDH), 4 U pyruvate kinase (PK), and 0.05% Triton X-100. The reaction was initiated by the addition of F6P, and NADH oxidation was monitored by the decrease in absorbance at 340 nm. PK activity was assessed using 1 μL of the sample in an assay buffer containing 100 mM KH_2_PO_4_, 10 mM MgCl_2_, 80 mM KCl, 0.05% Triton X-100, 9 U LDH, 0.17 mM NADH, and 5 mM ADP. The reaction was initiated by the addition of 2 mM PEP, and the change in absorbance was measured at 340 nm using UV-transparent microplates. LDH activity was determined using a commercial kit (LDH Liquiform, Labtest, Belo Horizonte, MG, Brazil), according to the manufacturer’s instructions. CS activity was measured using 2 μL of the sample added to a reaction mixture containing Tris-HCl, 1 mM MgCl_2_, 1 mM EDTA, 0.2 mM 5,5′-dithiobis(2-nitrobenzoic acid) (DTNB), and 0.3 mM acetyl-CoA. The reaction was initiated by the addition of 0.5 mM oxaloacetate, which was omitted from the control reaction. The absorbance change was monitored at 412 nm. CPT-1 activity was determined using 20 μL of the sample in a reaction buffer containing 60 mM Tris-HCl, 1.5 mM EDTA, 0.25 mM DTNB, 0.05% Triton X-100, and 0.035 mM palmitoyl-CoA (pH 8.1). The reaction was initiated by the addition of L-carnitine, and absorbance was measured at 412 nm. CK activity was assessed using 10 μL of the sample in an assay buffer (50 mM Tris-HCl, 2 mM MgSO_4_, 5 mM KCl, 0.2 mM NADH, 1 mM ATP, 10 mM creatine, 2 mM PEP, 2 U LDH, 4 U PK, and 0.05% Triton X-100). The reaction was initiated by the addition of creatine, and NADH oxidation was monitored by measuring the decrease in absorbance at 340 nm, using a 96-well microplate reader.

### 2.9. Statistical Analysis

The data are presented as the mean ± standard deviation or the median and interquartile range, according to distribution. Comparisons between the groups were performed using analysis of variance (ANOVA), followed by Tukey’s post hoc test or Kruskal–Wallis and Dunn’s test. The frequency of heart failure features was compared using Goodman’s test. The analyses were performed using Sigma Plot 12.0 (Systat Software Inc., Palo Alto, CA, USA). A significance level of 5% was adopted.

## 3. Results

### 3.1. Characterization of Experimental Groups and Frequency of Heart Failure Features

**Figure 1:**
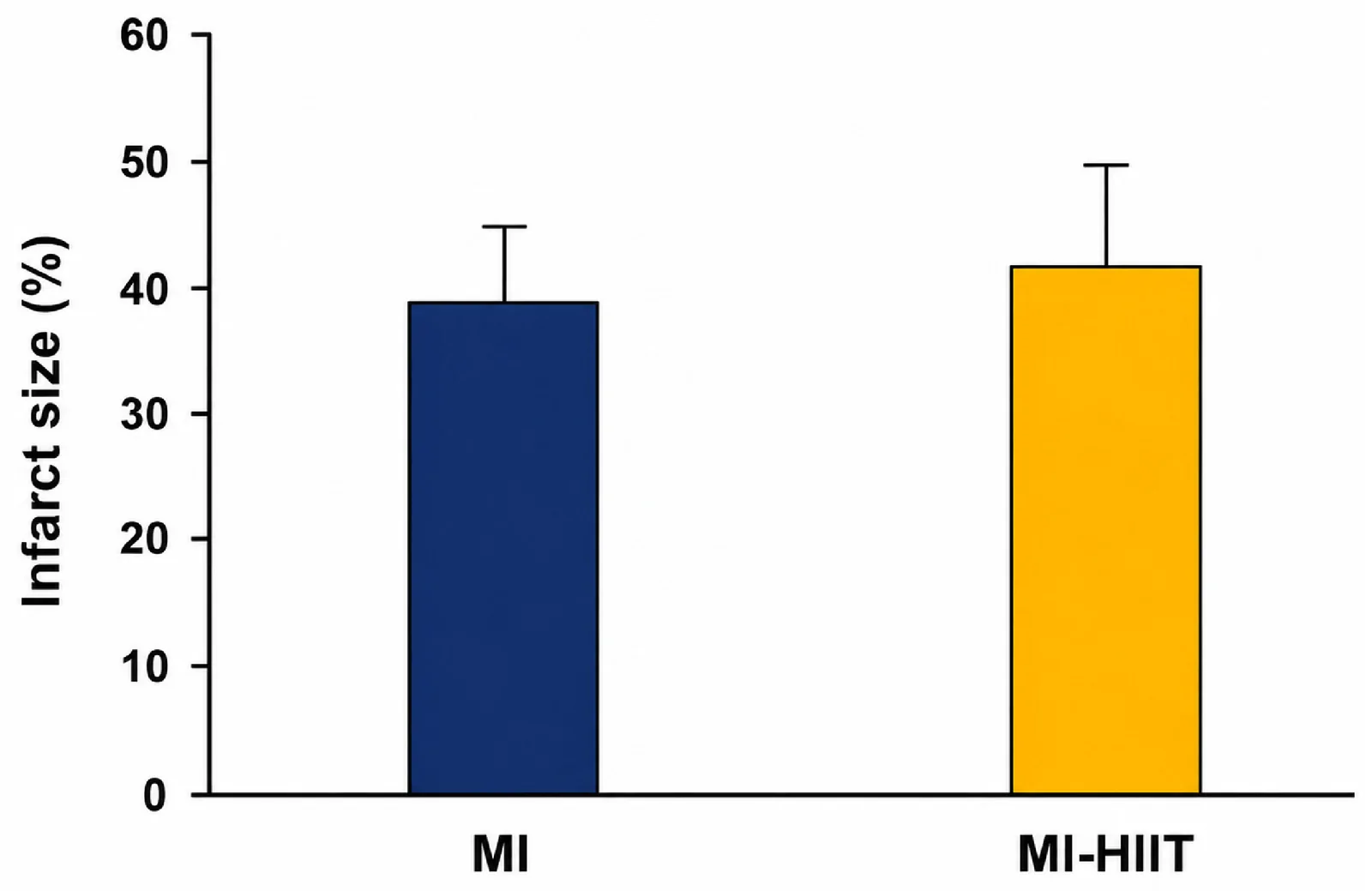
Histological myocardial infarct size as a percentage of total left ventricular (LV) area. Data are presented as mean ± standard deviation. MI: myocardial infarction (n = 9); MI-HIIT: MI subjected to high-intensity interval training (n = 9). Student’s *t*-test; *p* > 0.05.

To ensure homogeneous degrees of considerable cardiac injury, the rats with an infarct size less than 30% of the total LV area on histological analysis were excluded from this study. Thirteen animals were excluded from the MI group and nine from the MI-HIIT group. Four rats from the MI group and six from the MI-HIIT group died during the experiment; no deaths occurred in the Sham group. Thus, this study comprised 20 rats in the Sham group and nine in each of the MI and MI-HIIT groups. The frequency of heart failure features did not differ between the infarcted groups (Table 2). The infarct size analyzed by histomorphometry did not differ between the groups (Figure 1).

The initial and final body weights and left ventricular weight did not differ between the groups. The right ventricular weight normalized to body weight, atrial weight expressed as absolute values or normalized to body weight, and lung weight were higher in the infarcted groups than in the Sham group, with no differences observed between the MI and MI-HIIT groups (Table 3).

#### 3.1.1. Functional Capacity

**Figure 2:**
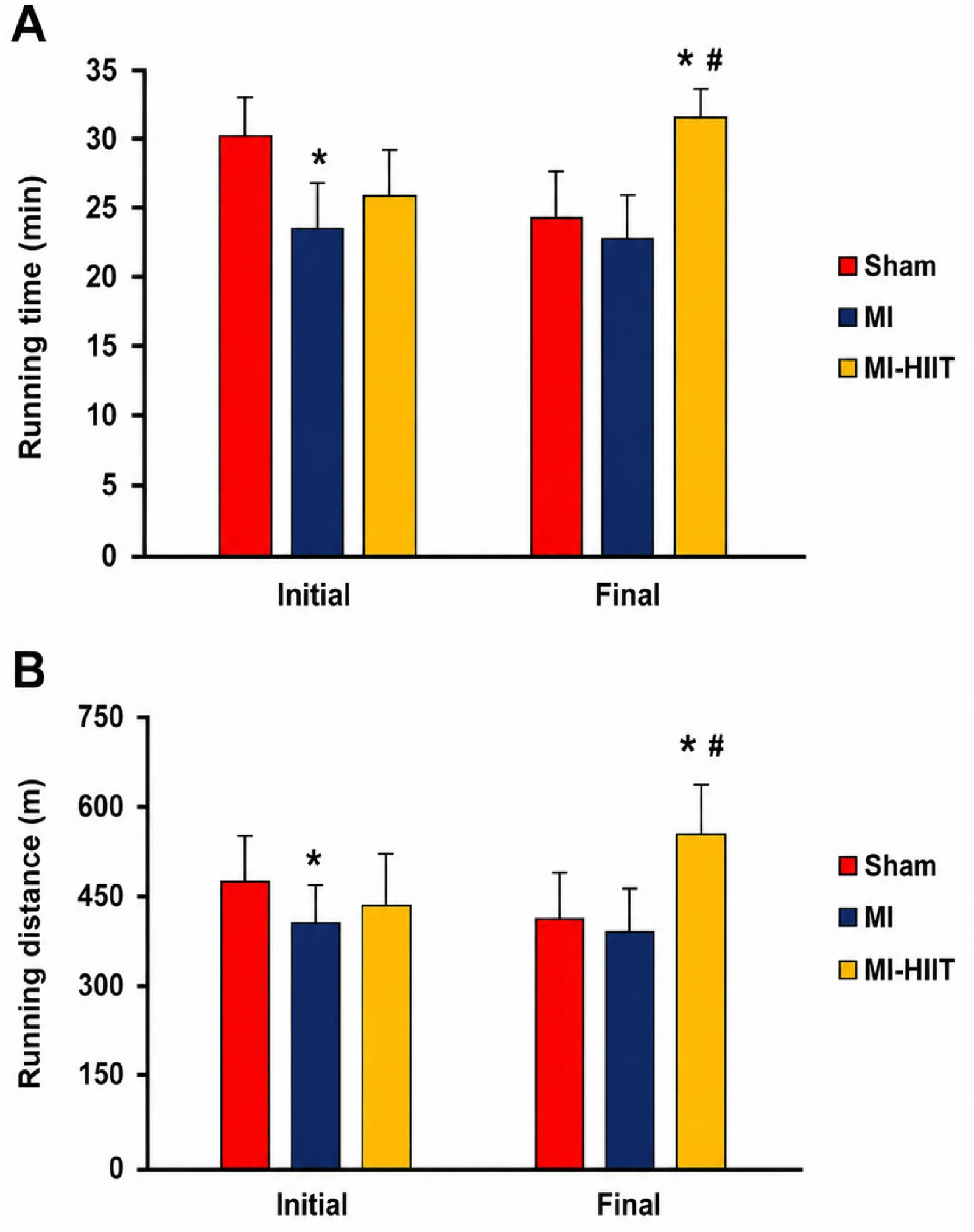
Functional capacity assessment. (**A**) Running time in the initial and final exercise tolerance tests. (**B**) Distance covered in the initial and final exercise tolerance tests. Data are expressed as mean ± standard deviation; Sham (n = 14); MI: myocardial infarction (n = 8); MI-HIIT: MI subjected to high-intensity interval training (n = 7). ANOVA and Tukey; * *p* < 0.05 vs. Sham; # *p* < 0.05 vs. MI.

The running time and distance covered in the exercise tolerance test performed before the exercise protocol were lower in the MI group than in the Sham group and did not differ between the MI-HIIT and MI groups. At the end of the experiment, functional capacity was higher in the MI-HIIT group than in the MI and Sham groups (Figure 2).

#### 3.1.2. Echocardiographic Assessment

The MI and MI-HIIT groups had left atrial dilation, LV hypertrophy, and systolic dysfunction when compared to Sham group, with no differences between the MI-HIIT and MI groups before training (Table 4, Table 5 and Table 6). The infarct size assessed by echocardiography did not differ between infarcted groups. At the end of the experiment, a similar pattern of cardiac chambers hypertrophy and dilation, and LV dysfunction was observed in the infarcted groups, with no differences between the MI-HIIT and MI groups (Table 7, Table 8 and Table 9).

#### 3.1.3. Muscle Trophism

**Figure 3:**
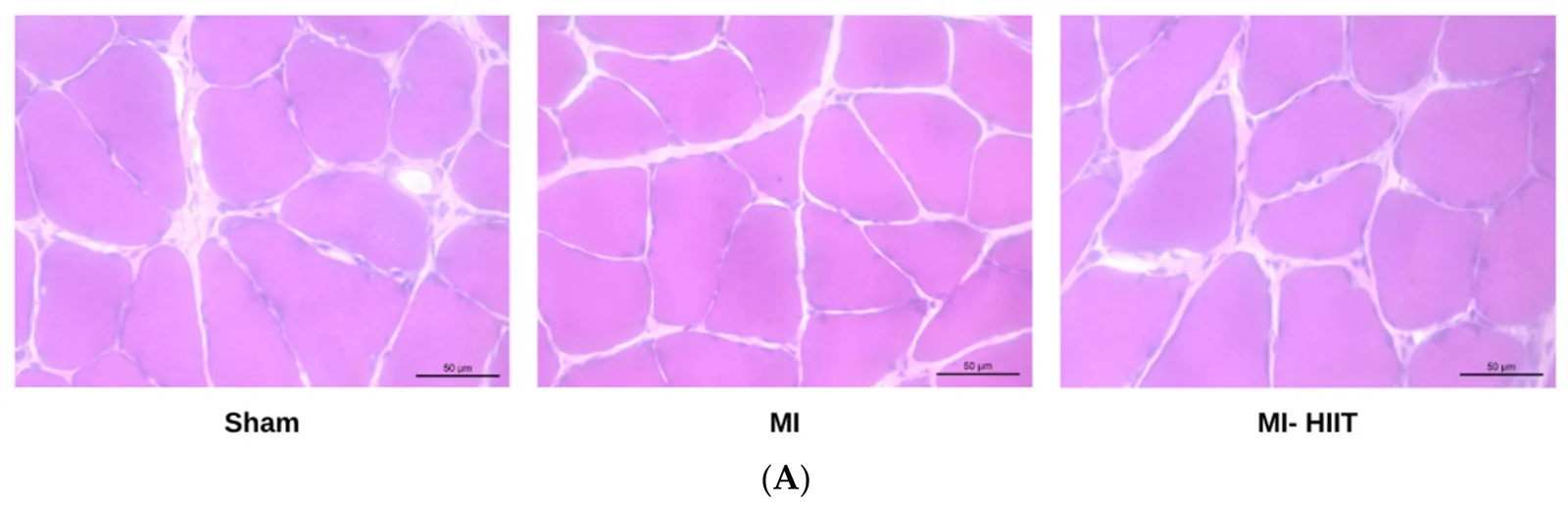

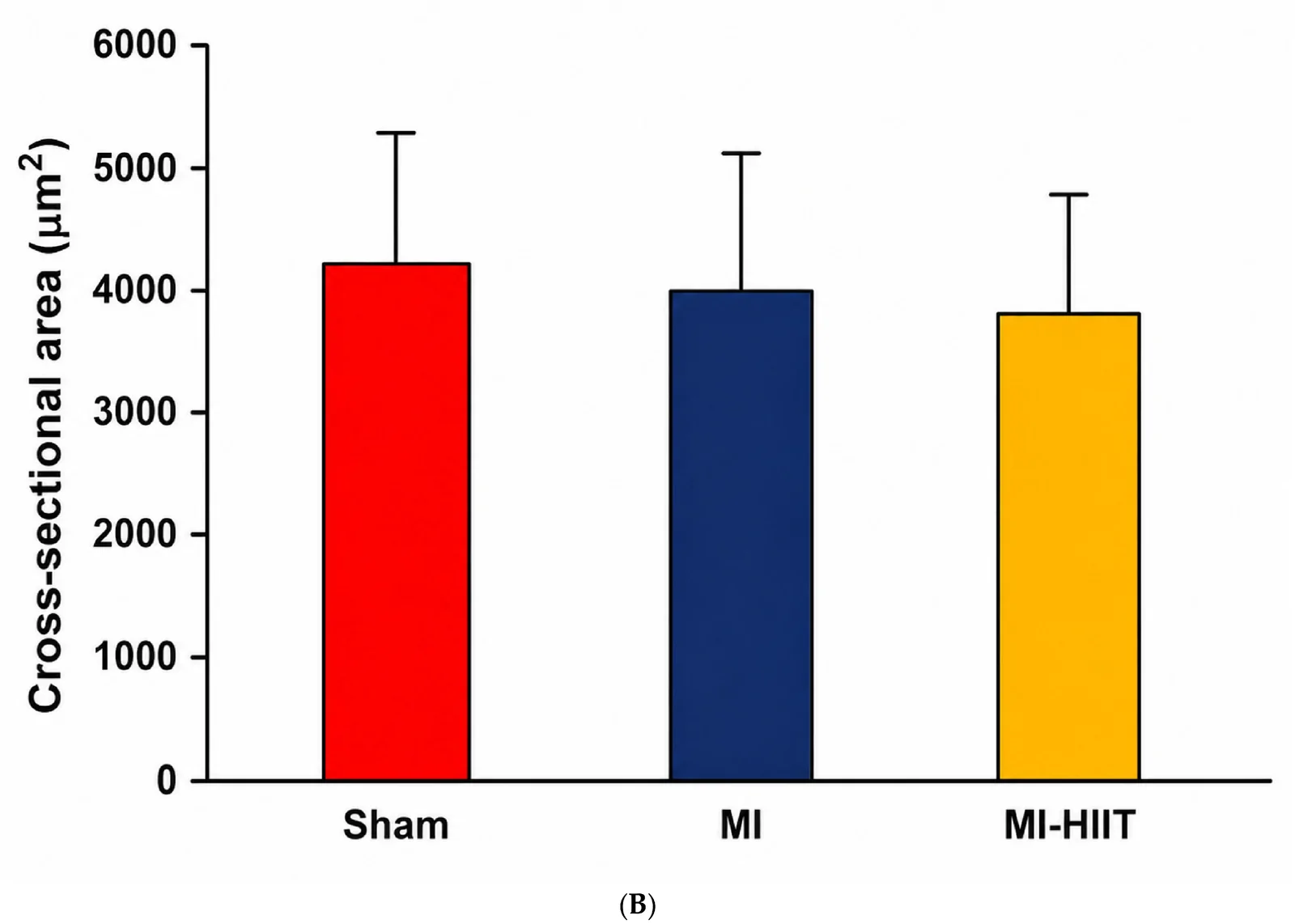
Cross-sectional area was assessed in hematoxylin and eosin-stained histological sections. (**A**): Representative images of soleus muscle sections. (**B**): Mean ± standard deviation. MI: myocardial infarction; MI-HIIT: MI subjected to high-intensity interval training; n = 8 rats per group; and scale bar = 50 μm. ANOVA and Tukey; *p* > 0.05.

The soleus cross-sectional area assessed in histological sections did not differ between groups (Figure 3).

#### 3.1.4. Energy Metabolism Enzyme Activities

Energy metabolism-related enzyme activities were unchanged in the MI group (Table 10). Pyruvate kinase and carnitine palmitoyltransferase 1 activities were higher in the MI-HIIT group than in the MI group, and citrate synthase was higher in the MI-HIIT group than in the Sham and MI groups.

#### 3.1.5. Oxidative Stress Assessment

Soleus lipid hydroperoxide levels and superoxide dismutase and glutathione peroxidase activities did not differ between groups. Catalase activity was lower in the MI and MI-HIIT groups than in the Sham group (Table 11).

## 4. Discussion

High-intensity interval training has been recommended as a non-pharmacological treatment for patients with different HF phenotypes, regardless of ejection fraction [25]. HIIT has been associated with changes in aerobic and anaerobic metabolic responses, and improvements in aerobic capacity and peripheral hemodynamics [26,27,28]. However, the effects of HIIT on skeletal muscle in long-term myocardial infarcted rats have not been established. This study showed, for the first time, that HIIT increases functional capacity and the activity of soleus muscle enzymes involved in energy metabolism despite no changes in cardiac remodeling and function.

Cardiac remodeling was induced by MI in rats. This model has been extensively used for studying the pathophysiology and treatment of cardiac remodeling and heart failure. This model has the advantages of being low cost, practical, and having good reproducibility when translating results to clinical studies. We have previously observed that only rats with large infarct sizes develop cardiac remodeling and LV dysfunction [29]. To ensure a considerable degree of cardiac injury, rats with an infarcted area < 30% of the total LV area were excluded from this study. Infarct size analyzed by echocardiogram did not differ between the groups. Infarct size is fully established 24 h after coronary artery ligation [29]. Therefore, both of the infarcted groups had similar degrees of cardiac injury prior to the training protocol. Histological assessment of the infarct size at the end of the experiment confirmed the similar infarct size between the groups.

Cardiac remodeling assessed in vivo by echocardiography before training showed left-side cardiac chambers dilation, LV hypertrophy, and LV systolic dysfunction. No differences were observed between the MI-HIIT and MI groups before training. Three months of HIIT did not modulate cardiac remodeling and LV function. At the end of the experiment, a similar pattern of left ventricular hypertrophy and dilation, and LV systolic dysfunction was observed in both infarcted groups, with no differences between the MI-HIIT and MI groups.

Despite exercise being recommended after MI, the most effective exercise modality and when to initiate exercise after MI remain controversial [25]. Studies evaluating the effects of HIIT on different cardiac remodeling models have shown disparate results in rodents [19,30,31]. Even so, most studies agree that higher exercise capacity is associated with reduced rehospitalization rates and improved quality of life in patients post-MI [28,32]. In this study, the HIIT protocol initiated three months after MI induction increased functional capacity compared with both Sham and MI groups. Similar results have been observed in previous studies. Functional capacity was higher in rats with small [33] or large [34] MI subjected to continuous moderate aerobic exercise compared with Sham and non-exercised infarcted rats. Therefore, our data show that late HIIT improves functional capacity in infarcted rats, despite unchanged cardiac remodeling and ventricular function. Thus, one possibility is that HIIT increases cardiorespiratory fitness by improving skeletal muscle function [12,14,35,36,37,38,39].

We, therefore, evaluated the peripheral skeletal muscle soleus. The cross-sectional area of muscle fibers did not differ between the groups, suggesting that infarcted rats did not progress morphologically to skeletal muscle atrophy, which is typically observed in advanced stages of clinical heart failure [40]. Heart failure-induced myopathy is described as a consequence of morphological, biochemical, and functional alterations at both local and systemic levels. These include inflammation, oxidative stress, mitochondrial dysfunction, increased peripheral vascular resistance due to sympathetic stimulation, endothelial dysfunction, interstitial fibrosis, and abnormalities in skeletal muscle oxygen use [41,42,43]. To explore the molecular events underlying the functional benefits induced by HIIT, we assessed skeletal muscle with a focus on oxidative stress and energy metabolism.

Oxidative stress is caused by an imbalance between reactive oxygen and nitrogen species production and antioxidant capacity. It leads to progressive damage in macromolecules and induces inflammation, fibrosis, alterations in intracellular calcium transients, and cell apoptosis [42,44]. Under physiological conditions, oxidative stress is neutralized by endogenous and exogenous antioxidant defenses. The main endogenous defenses are superoxide dismutase, catalase, and glutathione peroxidase [45]. Skeletal muscles consume large amounts of oxygen and may generate substantial quantities of reactive oxygen species, particularly during mitochondrial oxidative phosphorylation [46].

In this study, late HIIT training in post-MI rats did not reduce the oxidative stress marker lipid hydroperoxide concentration compared to the Sham and MI groups. Regarding antioxidant defenses, catalase activity was lower in the MI-HIIT and MI groups than in the Sham group, reinforcing the role of redox imbalance in the genesis and progression of ischemic heart failure [23,47,48]. While acute bouts of high-intensity exercise increase reactive oxygen species production [49], repeated exercise promotes adaptive increase in skeletal muscle antioxidant defenses through redox-sensitive pathways [48]. However, the magnitude and persistence of the response depend on the training modality, duration, and the interval between the last exercise session and tissue collection [49]. Thus, the absence of significant differences may reflect the chronic adaptive state of the trained muscle.

Changes in muscle metabolism markers have been reported in patients with HF [41]. Reduced concentrations of total cytosolic creatine kinase (CK), intramitochondrial citrate synthase, skeletal muscle-specific CK (MM-CK), and lactate dehydrogenase (LDH) were described in patients with HF with NYHA functional class III [50,51]. In this study, MI-HIIT had higher pyruvate kinase (PK), citrate synthase (CS), and carnitine palmitoyltransferase 1 (CPT-1) activities in the soleus compared to MI and Sham. These findings do not directly demonstrate enhanced mitochondrial respiration or ATP production. However, the increased activity of CPT-1 and PK, together with the higher CS activity, which are involved in glycolytic and oxidative metabolism, supports the presence of enhanced metabolic substrate utilization and oxidative capacity in the soleus muscle of infarcted rats following HIIT [50,52,53,54,55].

In this study, it was unexpected to find that HIIT did not improve cardiac function in long-term infarcted rats. As HIIT was initiated three months after MI, it is possible that the severe degree of cardiac dilation and LV dysfunction prevented a reverse remodeling process. Alterations in skeletal muscles have been observed during heart failure and are associated with reduced functional capacity [35]. Currently, no pharmacologic treatment is recommended for skeletal muscle changes [56]. Thus, it is important to identify strategies to prevent and treat muscle abnormalities. We showed that the improved peripheral skeletal muscle capacity was accompanied by the increased activity of soleus muscle enzymes involved in energy metabolism.

One limitation of this study is that we have not assessed muscle-fiber type composition, which could have added important information to our manuscript. Vescovo et al. [57] showed that changes in skeletal muscle composition may be involved in the functional capacity of patients with HF.

## 5. Conclusions

High-intensity interval training improves functional capacity despite unchanged cardiac remodeling and left ventricular function in long-term infarcted rats. Enhanced physical capacity is accompanied by increased activity of the enzymes involved in soleus muscle energy metabolism.

## Figures and Tables

**Table 1:** Adaptation to the exercise training protocol.

Weeks	Velocity (m/min)	Duration (min)
1	5	10
2	7.5	18
3	10–12	29
4	13–14	40
5	15–17	49

m: meters; min: minutes.

**Table 2:** Frequency (percentage and absolute number) of heart failure anatomopathological features.

	MI (n = 9)	MI-HIIT (n = 9)
Atria thrombus	11% (1)	–
Pleural effusion	11% (1)	22% (2)
Pulmonary congestion	44% (4)	22% (2)
RV hypertrophy	33% (3)	22% (2)
Ascites	22% (2)	33% (3)

MI: myocardial infarction; MI-HIIT: MI subjected to high-intensity interval training; n: number of rats; RV: right ventricle. Goodman’s test; *p* > 0.05.

**Table 3:** Anatomical variables.

	Sham (n = 20)	MI (n = 9)	MI-HIIT (n = 9)
BW (g)	532.19 ± 47.33	543.87 ± 62.84	517.69 ± 56.04
RV (g)	0.23 (0.21–0.26)	0.36 (0.29–0.47) *	0.26 (0.24–0.43)
RV/BW (mg/g)	0.45 (0.40–0.47)	0.71 (0.52–0.90) *	0.55 (0.47–0.74) *
Atria (g)	0.10 (0.09–0.12)	0.15 (0.11–0.31) *	0.21 (0.11–0.29) *
Atria/BW (mg/g)	0.20 (0.16–0.24)	0.32 (0.22–0.54) *	0.41 (0.22–0.56) *
Lung (g)	1.93 ± 0.62	2.98 ± 0.89 *	2.34 ± 0.59 *
Lung/BW (mg/g)	3.66 (3.27–3.86)	5.18 (4.03–7.35) *	3.94 (3.73–5.52)
Lung (W/D)	4.49 (4.38–4.54)	4.28 (3.89–4.87)	4.54 (4.15–4.78)
Soleus (g)	0.25 ± 0.02	0.23 ± 0.03	0.24 ± 0.04
Soleus/BW (mg/g)	0.47 ± 0.05	0.43 ± 0.07	0.47 ± 0.06

Data are expressed as mean ± standard deviation or median and percentiles. MI: myocardial infarction; MI-HIIT: MI subjected to high-intensity interval training; n: number of animals; BW: body weight; RV: right ventricle weight; W/D: ratio between wet-to-dry weight; Soleus: soleus weight. ANOVA and Tukey or Kruskal–Wallis and Dunn; * *p* < 0.05 vs. Sham.

**Table 4:** Baseline echocardiographic structural parameters.

	Sham (n = 20)	MI (n = 9)	MI-HIIT (n = 9)
BW (g)	462.93 ± 41.90	458.26 ± 42.17	447.88 ± 42.05
HR (bpm)	284.00 ± 25.62	295.07 ± 26.14	266.98 ± 30.64
LVEDD (mm)	8.07 ± 0.41	10.50 ± 0.69 *	9.82 ± 0.55 *
LVEDD/BW (mm/kg)	17.71 (16.14–18.53)	23.20 (21.14–25.23) *	21.48 (20.74–24.18) *
LV PWd (mm)	1.33 (1.27–1.37)	1.65 (1.53–1.96) *	1.63 (1.53–1.82) *
AO (mm)	4.01 (3.83–4.01)	3.94 (3.74–4.01)	3.83 (3.75–3.98)
LA (mm)	5.38 (5.16–5.66)	7.66 (6.66–8.51) *	6.59 (5.47–6.93)
LA/AO	1.36 (1.31–1.41)	1.94 (1.66–2.33) *	1.70 (1.41–1.82) *
LA/BW (mm/kg)	11.8 (10.5–12.7)	17.4 (13.9–19.6) *	14.7 (12.0–16.5)
LVM (g)	0.73 (0.66–0.80)	1.53 (1.21–1.75) *	1.25 (0.96–1.49) *
LVMI (g/kg)	1.56 (1.51–1.69)	3.58 (2.63–3.97) *	2.85 (2.15–3.17) *
LV RWT	0.33 ± 0.02	0.32 ± 0.04	0.34 ± 0.04
Infarct size (%)		41.20 ± 7.61	36.69 ± 6.69

Data are expressed as mean ± standard deviation or median and percentiles. MI: myocardial infarction; MI-HIIT: MI subjected to high-intensity interval training; n: number of rats; BW: body weight; HR: heart rate; bpm: beats per minute; LVEDD: left ventricular (LV) end-diastolic diameter; LV PWd: LV posterior wall thickness at end-diastole; AO: aorta diameter; LA: left atrial diameter; LVM: LV mass; LVMI: LVM index; LV RWT: LV relative wall thickness. ANOVA and Tukey or Kruskal–Wallis and Dunn; * *p* < 0.05 vs. Sham.

**Table 5:** Baseline echocardiographic assessment of left ventricular systolic function.

	Sham (n = 20)	MI (n = 9)	MI-HIIT (n = 9)
EFS (%)	56.04 ± 3.32	31.41 ± 5.78 *	31.78 ± 4.80 *
EF	0.86 (0.84–0.89)	0.52 (0.42–0.57) *	0.49 (0.44–0.57) *
PWSV (mm/s)	39.8 ± 5.4	24.5 ± 7.67 *	29.1 ± 7.15 *
Tei	0.45 (0.43–0.53)	0.74 (0.61–0.90) *	0.71 (0 56–0.80) *
FAC (%)	66.14 (63.68–69.19)	26.14 (20.17–37.37) *	34.18 (28.71–39.69) *
TDI S′ (average, cm/s)	3.47 ± 0.33	3.13 ± 0.36 *	2.98 ± 0.25 *

MI: myocardial infarction; MI-HIIT: MI subjected to high-intensity interval training; n: number of rats; EFS: endocardial fractional shortening; EF: ejection fraction; PWSV: posterior wall shortening velocity; Tei: myocardial performance index; FAC: fractional area change; TDI S′: tissue Doppler imaging of mitral annulus systolic velocity (average of lateral and septal walls). ANOVA and Tukey or Kruskal–Wallis and Dunn; * *p* < 0.05 vs. Sham.

**Table 6:** Baseline echocardiographic data of left ventricular diastolic function.

	Sham (n = 20)	MI (n = 9)	MI-HIIT (n = 9)
E/A	1.71 (1.49–1.86)	2.02 (1.38–5.55)	1.72 (1.39–1.95)
IVRT (ms)	26.00 (22.00–26.00)	30.00 (25.04–33.03)	30.01 (25.02–36.00) *
EDT (ms)	48.50 (45.00–56.00)	39.08 (35.52–46.33)	48.53 (42.54–59.50)
Mean E′ (cm/s)	4.15 ± 0.72	4.10 ± 0.52	3.49 ± 0.36 *
Lateral E′ (cm/s)	4.09 ± 0.81	4.33 ± 0.86	3.60 ± 0.69
Septal E′ (cm/s)	4.21 ± 0.74	3.86 ± 0.61	3.35 ± 0.31
Mean E/E′	18.79 (16.91–21.56)	24.08 (20.61–25.52) *	23.00 (20.82–23.90)
Mean A′ (cm/s)	2.90 (2.50–4.05)	2.85 (2.58–3.58)	2.75 (2.43–3.79)
Lateral A′ (cm/s)	3.20 (2.63–4.35)	3.00 (2.40–4.00)	2.70 (2.48–4.05)
Septal A′ (cm/s)	2.70 (2.40–3.08)	3.30 (2.40–4.50)	2.50 (2.33–3.65)
Mean E′/mean A′	1.38 ± 0.37	1.35 ± 0.47	1.20 ± 0.26

MI: myocardial infarction; MI-HIIT: MI subjected to high-intensity interval training; n: number of rats; E/A: ratio between early (E wave) and late (A wave) diastolic mitral inflow; IVRT: isovolumetric relaxation time; EDT: mitral E-wave deceleration time; E′: tissue Doppler imaging (TDI) of early mitral annulus diastolic velocity; A′: TDI of late mitral annulus diastolic velocity. Data are expressed as mean ± standard deviation or median and percentiles. ANOVA and Tukey or Kruskal–Wallis and Dunn; * *p* < 0.05 vs. Sham.

**Table 7:** Final echocardiographic assessment of cardiac structural parameters.

	Sham (n = 20)	MI (n = 9)	MI-HIIT (n = 9)
BW (g)	547.23 ± 38.09	550.23 ± 59.79	516.09 ± 58.64
HR (bpm)	268.98 ± 46.81	301.09 ± 32.38	308.89 ± 38.92
LVEDD	8.31 ± 0.41	11.10 ± 0.88 *	10.40 ± 0.98 *
LVESD (mm)	4.14 (3.96–4.30)	8.92 (8.32–9.65) *	8.59 (6.91–9.10) *
LVEDD/BW (mm/kg)	15.0 (14.4–16.3)	21.5 (18.5–22.2) *	20.6 (17.8–22.6) *
LV PWd (mm)	1.40 (1.38–1.45)	1.80 (1.63–2.03) *	1.72 (1.58–1.86) *
AO (mm)	4.20 ± 0.16	4.01 ± 0.23 *	4.07 ± 0.18
LA (mm)	5.66 (5.29–6.13)	8.76 (6.82–9.23) *	6.68 (6.05–9.26) *
LA/AO	1.37 (1.25–1.43)	2.22 (1.60–2.36) *	1.56 (1.45–2.35) *
LA/BW (mm/kg)	10.51 (9.22–11.14)	15.92 (11.78–17.56) *	13.89 (11.51–17.32) *
LVM (g)	0.85 (0.76–0.92)	1.66 (1.44–2.07) *	1.43 (1.27–1.74) *
LVMI (g/kg)	1.53 (1.46–1.70)	3.32 (2.63–4.11) *	3.00 (2.57–3.38) *
LV RWT	0.35 (0.33–0.36)	0.32 (0.30–0.36)	0.35 (0.30–0.37)
Infarct size (%)		44.00 (34.20–47.80)	37.90 (29.13–46.14)

Data are expressed as mean ± standard deviation or median and percentiles. MI: myocardial infarction; MI-HIIT: MI subjected to high-intensity interval training; n: number of rats; BW: body weight; HR: heart rate; bpm: beats per minute; LVEDD and LVESD: left ventricular (LV) end-diastolic and end-systolic diameters, respectively; LV PWd: LV posterior wall thickness at end-diastole; AO: aorta diameter; LA: left atrial diameter; LVM: LV mass; LVMI: LVM index; LV RWT: LV relative wall thickness. ANOVA and Tukey or Kruskal–Wallis and Dunn; * *p* < 0.05 vs. Sham.

**Table 8:** Final echocardiographic assessment of left ventricular systolic function.

	Sham (n = 20)	MI (n = 9)	MI-HIIT (n = 9)
EFS (%)	50.09 ± 3.58	19.78 ± 6.28 *	22.04 ± 5.19 *
EF	0.88 (0.85–0.90)	0.46 (0.40–0.55) *	0.49 (0.45–0.59) *
PWSV (mm/s)	41.14 ± 5.68	25.38 ± 9.35 *	31.82 ± 10.03 *
Tei	0.45 (0.42–0.50)	0.62 (0.52–0.69) *	0.62 (0.56–0.69) *
FAC (%)	68.50 ± 4.73	29.64 ± 8.92 *	34.28 ± 8.58 *
TDI S′ (average, cm/s)	3.55 ± 0.36	2.80 ± 0.49 *	2.98 ± 0.43 *

MI: myocardial infarction; MI-HIIT: MI subjected to high-intensity interval training; n: number of rats; EFS: endocardial fractional shortening; EF: ejection fraction; PWSV: posterior wall shortening velocity; Tei: myocardial performance index; FAC: fractional area change; TDI S′: tissue Doppler imaging of mitral annulus systolic velocity (average of lateral and septal walls). ANOVA and Tukey or Kruskal–Wallis and Dunn; * *p* < 0.05 vs. Sham.

**Table 9:** Final echocardiographic assessment of left ventricular diastolic function.

	Sham (n = 20)	MI (n = 9)	MI-HIIT (n = 9)
E/A	1.71 (1.36–1.82)	4.27 (1.27–6.12)	1.20 (1.08–4.31)
IVRT (ms)	26.00 (24.00–28.39)	24.00 (22.00–33.03)	26.00 (22.00–32.34)
EDT (ms)	51.03 (48.04–55.78)	33.04 (33.03–50.90) *	38.09 (32.23–46.32) *
Mean E′ (cm/s)	4.16 ± 0.68	4.18 ± 0.76	3.67 ± 0.39
Lateral E′ (cm/s)	4.24 ± 0.86	4.39 ± 1.06	3.46 ± 0.55
Septal E′ (cm/s)	4.09 ± 0.68	3.97 ± 1.22	3.89 ± 0.42
E/E′	19.20 ± 2.90	23.04 ± 3.35	24.78 ± 4.89 *
Mean A′ (cm/s)	3.67 ± 1.26	3.31 ± 1.35	3.93 ± 1.31
Lateral A′ (cm/s)	3.40 (2.75–4.65)	2.70 (2.20–2.90)	3.40 (2.20–5.40)
Septal A′ (cm/s)	3.58 ± 1.31	3.44 ± 1.31	4.01 ± 1.28
E′/A′	1.29 ± 0.54	1.38 ± 0.42	1.06 ± 0.46

MI: myocardial infarction; MI-HIIT: MI subjected to high-intensity interval training; n: number of rats; E/A: ratio between early (E wave) and late (A wave) diastolic mitral inflow; IVRT: isovolumetric relaxation time; EDT: mitral E-wave deceleration time; E′: tissue Doppler imaging (TDI) of early mitral annulus diastolic velocity; A′: TDI of late mitral annulus diastolic velocity. Data are expressed as mean ± standard deviation or median and percentiles. ANOVA and Tukey or Kruskal–Wallis and Dunn; * *p* < 0.05 vs. Sham.

**Table 10:** Activity of enzymes involved in energy metabolism.

	Sham (n = 8)	MI (n = 8)	MI-HIIT (n = 8)
CK	41.14 ± 4.96	40.51 ± 4.30	37.04 ± 13.9
PFK	16.20 (15.62–20.67)	20.56 (17.52–25.23)	19.67 (17.34–25.23)
PK	203.08 ± 32.02	186.79 ± 47.38	249.00 ± 40.58 #
LDH	495.07 ± 135.68	525.02 ± 55.91	540.09 ± 92.98
CS	66.04 ± 8.02	66.09 ± 10.12	81.28 ± 6.69 *#
CPT1	2.27 ± 0.60	2.35 ± 0.32	3.18 ± 0.67 #

MI: myocardial infarction; MI-HIIT: MI subjected to high-intensity interval training; n: number of rats; CK: creatine kinase; PFK: phosphofructokinase; PK: pyruvate kinase; LDH: lactate dehydrogenase; CS: citrate synthase; CPT1: carnitine palmitoyltransferase 1. Data are expressed as mean ± standard deviation or median and percentiles in nmol/min·mg protein. ANOVA and Tukey or Kruskal–Wallis and Dunn; * *p* < 0.05 vs. Sham; # *p* < 0.05 vs. MI.

**Table 11:** Oxidative stress markers.

	Sham (n = 8)	MI (n = 8)	MI-HIIT (n = 8)
HP (nmol/g tissue)	106.09 ± 16.52	121.43 ± 12.90	98.98 ± 26.72
SOD (nmol/mg protein)	16.62 (15.78–17.67)	18.4 (15.98–20.34)	15.52 (14.51–17.9)
Catalase (µmol/g tissue)	55.99 ± 4.95	39.82 ± 10.03 *	31.70 ± 5.25 *
GPX (nmol/mg tissue)	169.07 ± 33.04	126.17 ± 27.49	145.89 ± 41.73

MI: myocardial infarction; MI-HIIT: MI subjected to high-intensity interval training; n: number of rats; HP: lipid hydroperoxide; SOD: superoxide dismutase; GPx: glutathione peroxidase. Data are expressed as mean ± standard deviation or median and percentiles. ANOVA and Tukey or Kruskal–Wallis and Dunn; * *p* < 0.05 vs. Sham.

## Data Availability

All data generated or analyzed during this study are included in this manuscript.

## Abbreviations

The following abbreviations were used in this manuscript:

HIIT	High-intensity interval training
MI	Myocardial infarction
VO2 peak	Peak oxygen uptake
LV	Left ventricle
HE	Hematoxylin and eosin
ANOVA	Analysis of variance
CK	Creatine kinase
MM- CK	Skeletal muscle-specific creatine kinase
LDH	Lactate dehydrogenase
NYHA	New York Heart Association
PK	Pyruvate kinase
CS	Citrate synthase
CPT-1	Carnitine palmitoyltransferase 1
F6P	Fructose-6-phosphate
PEP	Phosphoenolpyruvate
